# Barriers to Lynch Syndrome Testing and Preoperative Result Availability in Early-onset Colorectal Cancer: A National Physician Survey Study

**DOI:** 10.1038/s41424-018-0047-y

**Published:** 2018-09-20

**Authors:** Alan Noll, Parth J. Parekh, Meijiao Zhou, Thomas K. Weber, Dennis Ahnen, Xiao-Cheng Wu, Jordan J. Karlitz

**Affiliations:** 1 Tulane University School of Medicine, New Orleans, LA, USA;, Emory University School of Medicine, Atlanta, GA USA; 2 Tulane University School of Medicine, New Orleans, LA, USA;, Eastern Virginia Medical School, Norfolk, VA USA; 3 Louisiana Tumor Registry and Epidemiology, School of Public Health, Louisiana State University Health Sciences Center, New Orleans, LA USA; 4 Zucker School of Medicine, Hofstra Northwell, Northwell Health Cancer Institute, Hempstead, NY USA; 5 Gastroenterology of the Rockies, University of Colorado School of Medicine, Aurora, CO USA; 6 Tulane University School of Medicine, Division of Gastroenterology. Staff Gastroenterologist Southeast Louisiana Veteran Health Care System, New Orleans, LA USA

## Abstract

**Objective:**

Although widely recommended, Lynch syndrome (LS) testing with tumor microsatellite instability (MSI) and/or immunohistochemistry (IHC) is infrequently performed in early-onset colorectal cancer (CRC), and CRC generally. Reasons are poorly understood. Hence, we conducted a national survey focusing on gastroenterologists, as they are frequently first to diagnose CRC, assessing testing barriers and which specialist is felt responsible for ordering MSI/IHC. Additionally, we assessed factors influencing timing of MSI/IHC ordering; testing on colonoscopy biopsy, opposed to post-operative surgical specimens, assists decisions on preoperative germline genetic testing and extent of colonic resection (ECR).

**Methods:**

A 21-question web-based survey was distributed through an American College of Gastroenterology email listing.

**Results:**

In total 509 completed the survey. 442 confirmed gastroenterologists were analyzed. Only 33.4% felt gastroenterologists were responsible for MSI/IHC ordering; pathologists were believed most responsible (38.6%). Cost, unfamiliarity interpreting results and unavailable genetic counseling most commonly prevented routine ordering (33.3%, 29.2%, 24.9%, respectively). In multivariable analysis, non-academic and rural settings were associated with cost and genetic counseling barriers. Only 46.1% felt MSI/IHC should always be performed on colonoscopy biopsy. Guideline familiarity predicted whether respondents felt surgical resection should be delayed until results returned given potential effect on ECR decisions.

**Conclusion:**

Inconsistencies in who is felt should order MSI/IHC may lead to diffusion of responsibility, preventing consistent testing, including preoperatively. Assuring institutional universal testing protocols are in place, with focus on timing of testing, can optimize care. Strategies addressing cost barriers and genomic service availability in rural and non-academic settings can enhance testing. Greater emphasis on guideline familiarity is required.

## Introduction

CRC is the third most common cancer in men and women^1^. It is the 3rd leading cause of cancer death in women and 2nd in men. Up to 4% of CRCs are attributed to Lynch Syndrome (LS), with penetrance for CRC development as high as 82%^2,3^. In early-onset cases, LS can be common with up to 17% unselected for family history being diagnosed^4–7^.

Guidelines recommend testing all CRCs, regardless of patient age, for microsatellite instability (MSI) and/or with immunohistochemistry (IHC) for mismatch repair proteins (MLH1, MSH2, MSH6, PMS2) as initial screen for LS^2,8–10^. Ideally, results should be available preoperatively to help facilitate surgical planning^11^. Studies demonstrate IHC on preoperative colonoscopic biopsy specimens correlates well with surgical resection specimens^12,13^. Abnormal MSI/IHC prompts germline genetic testing to identify a mutation, confirming LS.

LS identification is important as surveillance colonoscopy decreases mortality^14^. Additionally, extra-colonic cancer screening is performed^2^. Preoperative LS identification affects operative decisions regarding extended colonic resection (ECR). Observational studies demonstrate ECR (subtotal/total colectomy) decreases metachronous cancer development and can increase life expectancy, particularly in younger patients^15–17^. One study demonstrated with segmental resection, metachronous rates may be 16% at 10 years, 41% at 20 years and 62% at 30 years post-operatively, even with frequent post-operative colonoscopic surveillance, versus 0% in extended colectomy^15^. Hence, guidelines recommend ECR for cancer risk reduction^18,19^. Finally, LS identification guides germline testing in family members.

Despite benefits, MSI/IHC is infrequently performed. In a statewide population-based study of early-onset CRC patients, MSI and/or IHC was only performed in 23%^20^. Of those tested, results were available preoperatively in only 16.9%. This was felt to contribute to low subtotal/total colectomy rates, even with abnormal MSI and/or IHC, in this same early-onset population^21^. Low mismatch repair deficiency testing was recently demonstrated nationally with only 28.2% of CRC patients overall and 43.1% of early-onset patients tested^22^. Reasons for inadequate testing have not been well studied, but potentially include decreased understanding of testing importance, effect on management, unclear responsibility regarding who should order testing (gastroenterologist, pathologist, etc.) and lack of specialists (i.e., genetic counselors) to assist with testing/interpretation.

To explore LS testing practices and barriers, we conducted a national survey of physicians in 2017, the overwhelming majority of whom were gastroenterologists. Gastroenterologists are important as they frequently first diagnose CRC and can facilitate preoperative LS identification by helping assure MSI/IHC is performed on colonoscopy biopsy specimens. Although testing is recommended in patients of all ages, the survey focuses on early-onset CRC patients as they are often at highest risk of hereditary syndromes. Hence studying testing practices in this group may provide a best-case management scenario.

We asked the following questions.

1) Which specialist in the cancer care continuum do study participants feel is responsible for ordering MSI/IHC?

2) What barriers prevent ordering MSI/IHC?

3) What factors affect preoperative result availability?

4) In LS patients, what operative approach, ECR vs. segmental resection, is felt should be taken?

## Methods

### Survey overview

The Tulane Institutional Review Board approved this study. Study investigators with expertise in hereditary cancer syndromes developed survey questions (Supplemental Fig. 1). An opportunity to enter a $25 gift card raffle was given. Web-based survey items were presented in scenario/non-scenario formats using Qualtrics software.

The survey was distributed March 2017 through an American College of Gastroenterology (ACG) email listing. Reminders were sent 2 weeks later. Emails came from the ACG directly, including content explanation and embedded URL survey links. Submissions closed 4 weeks after the 2nd email. Given that some ACG members may be non-gastroenterologists, specific questions were also developed for surgeons, pathologists and other providers. However, given low responses from non-gastroenterologists, they were ultimately excluded from analysis.

### Statistical analysis

Frequency tables were created for demographics and responses to decision-making questions. 10-year career stage intervals were chosen based on board certification/recertification intervals. For questions with multiple responses, comparisons were made using meaningfully pooled data. Multivariable logistic regression was employed to assess the association of responses with demographic factors. Chi-squared analysis was utilized. Odds ratios were reported with 95% confidence intervals. Analyses were performed using SAS 9.4 with p-values of 0.05 considered significant.

## Results

### Response rate

The survey link was distributed to 11,924 email addresses. Emails were opened, confirming receipt, in 4491 instances with links followed in 320 instances. Reminders were distributed 2 weeks later, with 3,508 opening it and 183 following the link. There were 5786 unique confirmed email recipients (2213 opened both the first and 2nd emails) yielding an overall response rate of 8.7%. Confirmed email receipts were used to calculate response rate versus all distribution emails as many accounts have potential to be out-of-date, infrequently used or members may potentially have multiple email addresses listed. The response rate was 4.2% if using the entire email list. Response rates to individual questions/items were >80% in all cases (80.6–97.7%).

In total 509 participants took the survey. 6 of 509 accessed surveys without receiving the ACG email directly. In total 505 answered whether they were a medical student or resident. 31 students/residents, 13 non-gastroenterologists and 19 respondents who never indicated their medical specialty were excluded (442 respondents analyzed).

### Participant demographics

71.3% identified as general gastroenterologists, followed by advanced endoscopy and other subspecialties (Table 1). 85.6% practiced in urban counties. The most common practice settings were “single-specialty private practice” and “university/academic” (40.6%, 31.1% respectively). 52.0% were in practice >10 years, 33.9% between 0 and 10 years and 14.2% were in fellowship.

**Table 1:** Demographics of survey respondents (*N* = 442)

Demographic variable	Frequency	Percentage
Gastroenterology subspecialty		
General GI	308	71.3
GI Oncology^a^	19	4.4
Hepatology	20	4.6
Inflammatory Bowel Disease (IBD)	35	8.1
Functional/Motility	5	1.2
Advanced Endoscopy	45	10.4
Urban/rural location^b^		
Urban	374	85.6
Rural	63	14.4
Practice setting		
Multispeciality Private Practice	57	13.0
Hospital Employed	52	11.9
Single Specialty Private Practice	178	40.6
University/Academic Center	136	31.1
Veterans Affairs	15	3.4
Career Stage		
Fellows in training	61	14.2
In practice from 0 to 10 years	146	33.9
In practice from 11 + years	224	52.0

^a^GI Oncology was presented as a sub specialization to those respondents who previously identified as gastroenterologists to avoid confusion with medical oncologists

^b^This study defines rural versus urban areas based upon the USDA’s 2013 “Rural-Urban Continuum Codes,” a classification scheme that distinguishes metro counties by population size and non-metro, or rural, areas by degree of urbanization and adjacency to metro areas. Survey takers were provided access to the continuum coded spreadsheet with instructions to help define the county in which they practiced

### Specialist felt responsible for ordering MSI/IHC

**Fig. 1:**
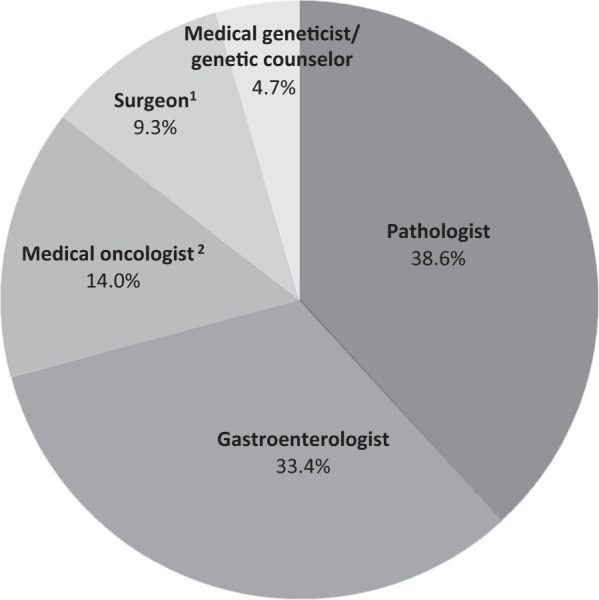
Healthcare provider believed to be responsible for ordering MSI/IHC testing to screen for Lynch Syndrome in newly diagnosed CRC under the age of 50. ^1^For the purposes of this study, “Surgeon” refers to Colorectal Surgeon, General Surgeon, or Surgical Oncologist. ^2^“Medical Oncologist” refers to those physicians who have completed an Internal Medicine residency followed by a Hematology/Oncology Fellowship. Gastroenterologists who specialize in genetics and GI cancers are included above in the “Gastroenterologist” category

No consensus exists among gastroenterologists regarding which specialist they feel should be responsible for ordering MSI/IHC (Fig. 1). Pathologists were believed most responsible (38.6%) followed by gastroenterologists (33.4%). Medical oncologists, surgeons and geneticists were felt to be responsible less frequently.

### Barriers to MSI/IHC test ordering

Cost, unfamiliarity interpreting results and unavailable genetic counseling were the most common reasons preventing testing, Table 2 (22.4%, 18.5%, 15.9%, respectively). In multivariable analysis, non-academic or rural settings and being a GI fellow were associated with a higher perception of deficiencies in access to genetic counseling and germline testing and belief MSI/IHC cost was prohibitive. GI fellows, those in early-career stages and those in non-academic settings more commonly believed waiting for MSI/IHC results would delay colon resection and therefore negatively impact cancer outcomes, thus preventing initial ordering. Those in non-academic settings also felt waiting for germline testing results (if MSI/IHC were abnormal) would delay colonic resection and negatively impact outcomes.

**Table 2:** Barriers to ordering MSI/IHC testing, multivariable analysis

Barriers to MSI/IHC Testing Ordering (Number of respondents indicating this as a barrier/total respondents answering this question; percentage)	Variables	Number of respondents answering yes (Percent respondents)	Odds ratio (95% Confidence interval)
Cost of MSI and/or IHC testing is prohibitive *(85/379; 22.4%)*	Gastroenterology subspecialty		
	General GI	58 (21.5)	1.00
	Non-general GI	**27 (25.7)**	**1.96 (1.07, 3.60)**
	Urban/rural location		
	Urban	65 (20.5)	1.00
	Rural	**20 (33.9)**	**1.95 (1.01, 3.79)**
	Practice Setting		
	Academic Center^a^	19 (17.9)	1.00
	Non-academic Center	**66 (24.5)**	**2.55 (1.24, 5.23)**
	Career Stage		
	Fellows in training	**14 (29.8)**	**2.97 (1.27, 6.93)**
	In practice from 0 to 10 years	**34 (26.6)**	**1.82 (1.02, 3.23)**
	In practice from 11+ years	36 (17.9)	1.00
	Familiarity with the guideline		
	Familiar	29 (17.9)	1.00
	Unsure^b^	25 (20.8)	1.29 (0.70, 2.39)
	Unfamiliar^c^	**27 (32.9)**	**2.05 (1.08, 3.88)**
Lack of familiarity interpreting and applying the results from MSI and/or IHC testing *(70/379; 18.5%)*	Gastroenterology subspecialty		
	General GI	54 (20.0)	1.00
	Non-general GI	16 (15.2)	0.81 (0.40, 1.61)
	Urban/rural location		
	Urban	54 (17.0)	1.00
	Rural	15 (25.4)	1.41 (0.69, 2.88)
	Practice Setting		
	Academic Center^a^	19 (18.1)	1.00
	Non-academic Center	51 (18.9)	1.13 (0.54, 2.35)
	Career Stage		
	Fellows in training	13 (27.7)	1.99 (0.83, 4.80)
	In practice from 0 to 10 years	23 (18.0)	1.24 (0.66, 2.31)
	In practice from 11+years	33 (16.4)	1.00
	Familiarity with the guideline		
	Familiar	17 (10.6)	1.00
	Unsure^b^	**23 (18.9)**	**2.04 (1.03, 4.04)**
	Unfamiliar^c^	**28 (34.6)**	**3.89 (1.94, 7.80)**
Lack of access to genetic counseling at my facility *(60/378; 15.9%)*	Gastroenterology subspecialty		
	General GI	50 (18.5)	1.00
	Non-general GI	10 (9.6)	0.74 (0.33, 1.67)
	Urban/rural location		
	Urban	42 (13.3)	1.00
	Rural	**18 (30.0)**	**2.22 (1.09, 4.50)**
	Practice Setting		
	Academic Center^a^	6 (5.7)	1.00
	Non-academic Center	**54 (20.2)**	**6.96 (2.31, 20.94)**
	Career Stage		
	Fellows in training	**12 (25.5)**	**5.70 (2.10, 15.51)**
	In practice from 0 to 10 years	17 (13.3)	1.08 (0.54, 2.14)
	In practice from 11+ years	30 (15.0)	1.00
	Familiarity with the guideline		
	Familiar	20 (12.3)	1.00
	Unsure^b^	19 (15.8)	1.43 (0.70, 2.93)
	Unfamiliar^c^	**20 (25.0)**	**2.16 (1.03, 4.53)**
Lack of access to germline genetic testing if MSI/IHC abnormal *(51/381; 13.4%)*	Gastroenterology (GI) subspecialty		
	General GI	41 (15.1)	1.00
	Non-general GI	10 (9.5)	0.88 (0.39, 2.00)
	Urban/rural location		
	Urban	35 (11.0)	1.00
	Rural	**16 (27.1)**	**2.61 (1.26, 5.41)**
	Practice Setting		
	Academic Center^a^	7 (6.6)	1.00
	Non-academic Center	**44 (16.2)**	**3.68 (1.32, 10.22)**
	Career Stage		
	Fellows in training	**9 (18.8)**	**3.14 (1.15, 8.63)**
	In practice from 0 to 10 years	18 (14.1)	1.61 (0.80, 3.25)
	In practice from 11+ years	23 (11.4)	1.00
	Familiarity with the guideline		
	Familiar	17 (10.4)	1.00
	Unsure^b^	17 (13.9)	1.41 (0.67, 2.96)
	Unfamiliar^c^	15 (18.5)	1.60 (0.72, 3.55)
Waiting for germline testing results (after initial MSI and/or IHC testing) would delay resection and therefore negatively impact the patient’s outcome *(45/376; 12.0%)*	Gastroenterology subspecialty		
	General GI	37 (13.8)	1.00
	Non-general GI	8 (7.7)	0.71 (0.29, 1.75)
	Urban/rural location		
	Urban	36 (11.4)	1.00
	Rural	9 (15.8)	1.16 (0.49, 2.74)
	Practice Setting		
	Academic Center^a^	7 (6.7)	1.00
	Non-academic Center	**38 (14.2)**	**3.91 (1.31, 11.66)**
	Career Stage		
	Fellows in training	7 (14.9)	2.70 (0.90, 8.07)
	In practice from 0 to 10 years	17 (13.3)	1.83 (0.89, 3.75)
	In practice from 11+ years	20 (10.1)	1.00
	Familiarity with the guideline		
	Familiar	17 (10.6)	1.00
	Unsure^b^	16 (13.3)	1.36 (0.64, 2.86)
	Unfamiliar^c^	10 (12.5)	1.03 (0.43, 2.48)
Waiting for MSI and/or IHC testing results would delay colon resection and therefore negatively impact the patient’s outcome (35/378; 9.3%)	Gastroenterology subspecialty		
	General GI	30 (11.2)	1.00
	Non-general GI	5 (4.8)	0.52 (0.17, 1.61)
	Urban/rural location		
	Urban	27 (8.5)	1.00
	Rural	8 (13.6)	1.36 (0.53, 3.48)
	Practice Setting		
	Academic Center^a^	5 (4.7)	1.00
	Non-academic Center	**30 (11.2)**	**5.38 (1.39, 20.83)**
	Career Stage		
	Fellows in training	**6 (12.8)**	**4.23 (1.23, 14.54)**
	In practice from 0 to 10 years	**15 (11.7)**	**2.78 (1.22, 6.34)**
	In practice from 11+ years	13 (6.5)	1.00
	Familiarity with the guideline		
	Familiar	10 (6.2)	1.00
	Unsure^b^	15 (12.5)	2.29 (0.96, 5.45)
	Unfamiliar^c^	8 (9.9)	1.36 (0.48, 3.84)
Ordering Testing may adversely affect a patient’s medical insurance status (35/376; 9.3%)	Gastroenterology subspecialty		
	General GI	28 (10.4)	1.00
	Non-general GI	7 (6.8)	0.84 (0.33, 2.13)
	Urban/rural location		
	Urban	27 (8.6)	1.00
	Rural	8 (13.8)	1.39 (0.55, 3.47)
	Practice Setting		
	Academic Center^a^	8 (7.6)	1.00
	Non-academic Center	27 (10.1)	1.60 (0.58, 4.41)
	Career Stage		
	Fellows in training	6 (12.8)	1.76 (0.54, 5.67)
	In practice from 0 to 10 years	12 (9.5)	1.38 (0.62, 3.08)
	In practice from 11+ years	16 (8.0)	1.00
	Familiarity with the guideline		
	Familiar	12 (7.4)	1.00
	Unsure^b^	11 (9.2)	1.30 (0.55, 3.07)
	Unfamiliar^c^	11 (13.8)	1.70 (0.70, 4.18)

^a^Academic center includes university/academic center; and non-academic center incluldes multispeciality private practice, hospital employed physician, single specialty private practice, and veterans affairs facility

^b^Unsure means they are not clear on whether they are aware of the applicable guidelines or not

^c^Unfamiliar means providers are not well versed with current guidelines

When barriers were stratified based on routine versus non-routine test ordering on colonoscopy, all became magnified (Table 3). Most common barriers in non-routine orderers included cost (33.3%), lack of familiarity interpreting/applying results (29.2%) and decreased genetic counseling access (24.9%).

**Table 3:** Barriers to MSI/IHC ordering stratified by those routinely and non-routinely ordering testing on colonoscopy biopsy specimens

Barrier to MSI/IHC test ordering	Non-routinely ordering^a^	Routinely ordering^a^	Odds ratio (95% CI)
Cost of MSI and/or IHC testing is prohibitive	33.3%	11.7%	3.79 (2.19, 6.53)
Lack of familiarity interpreting and applying the results from MSI and/or IHC testing	29.2%	6.2%	6.23 (3.15, 12.35)
Lack of access to genetic counseling at my facility	24.9%	5.6%	5.56 (2.72, 11.39)
Lack of access to germline genetic testing if MSI/IHC abnormal	20.0%	6.2%	3.82 (1.89, 7.72)
Waiting for germline testing results (after initial MSI and/or IHC testing) would delay resection and therefore negatively impact the patient’s outcome	18.2%	5.7%	3.72 (1.78, 7.77)
Waiting for MSI and/or IHC testing results would delay colon resection and therefore negatively impact the patient’s outcome	14.1%	4.5%	3.50 (1.54, 7.92)
Ordering testing may adversely affect a patient’s medical insurance status	12.0%	6.2%	2.08 (0.98, 4.40)

^a^The definition of routinely vs. non-routinely ordering is based on the survey question of “What percentage of the time will you plan to perform MSI and/or IHC testing for LS on tumor biopsies taken during colonoscopy?” Non-routinely ordering are the respondents who answer “0%”, “25%”, “50%” or “75%” of the time, and the routinely ordering are those respondents who answer “100%” of the time

### Timing of MSI and/or IHC testing

**Fig. 2:**
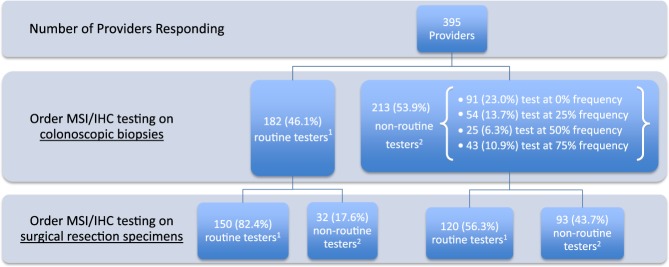
MSI/IHC ordering practices on colonoscopy biopsy and surgical resection specimens. ^1^“Routine tester” is defined as a provider indicating a frequency of ordering MSI/IHC testing in 100% of cases of CRC in patients < 50 years old. This definition applies to both testing performed on colonoscopic biopsies or surgical resection specimens. ^2^ “Non-routine tester” is defined as a provider indicating a frequency of ordering MSI/IHC testing in “0%”, “25%”, “50%” or “75%” of the time in CRC patients < 50 years old. This definition likewise applies to both testing performed on colonoscopic biopsies or surgical resection specimens

Only 46.1% felt MSI/IHC should be routinely (100% of the time) performed on colonoscopy biopsy specimens (Fig. 2). In the 53.9% that do not routinely (0–75% of the time) order MSI/IHC on colonoscopy biopsy, 43.7% also do not believe that MSI/IHC should be performed routinely on surgical resection specimens. In those routinely performing MSI/IHC on colonoscopy biopsy, 82.4% believe testing should also be performed concurrently on surgical resection specimens. Participants familiar with guidelines, in practice from 0 to 10 years, GI subspecialists (including GI oncology) and those working in academic or urban settings were most likely to believe testing should be performed on preoperative colonoscopy biopsy (Table 4).

**Table 4:** Analysis of subgroup opinions regarding routine performance of MSI/IHC testing on colonoscopy biopsy specimens, surgical resection specimens or both

Performance of MSI/IHC testing	Gastroenterologist Subgroups		Number of participants who answered “100% of the time” (%)
Will plan to perform MSI/IHC testing on PRE-SURGICAL tumor biopsies taken during colonoscopy	Gastroenterology (GI) subspecialty		
		General GI	125 (43.4)
		GI Oncology	10 (55.6)
		All other GI Specializations	55 (55.6)
	Urban/rural location		
		Urban	172 (50.0)
		Rural	19 (31.2)
	Practice setting		
		Academic Center	64 (52.5)
		Non-academic Center	127 (44.9)
	Career stage		
		Fellows in training	25 (47.2)
		In practice 0–10 years	75 (54.7)
		In practice 11+ years	91 (42.5)
	Familiarity with the guideline		
		Familiar	86 (53.8)
		Unsure^a^	58 (46.8)
		Unfamiliar^b^	30 (36.1)
Expect MSI/IHC testing performed on POST-SURGICAL specimens	Gastroenterology (GI) subspecialty		
		General GI	186 (65.3)
		GI Oncology	17 (89.5)
		All other GI Specializations	74 (76.3)
	Urban/rural location		
		Urban	240 (70.6)
		Rural	36 (59.0)
	Practice setting		
		Academic Center	86 (72.9)
		Non-academic Center	192 (67.6)
	Career stage		
		Fellows in training	32 (59.3)
		In practice 0–10 years	90 (66.2)
		In practice 11+ years	155 (73.5)
	Familiarity with the guideline		
		Familiar	115 (73.3)
		Unsure^a^	81 (67.5)
		Unfamiliar^b^	48 (57.1)
Plan to perform MSI/IHC testing both on colonoscopy and post-surgical specimens	Gastroenterology (GI) subspecialty		
		General GI	92 (33.2)
		GI Oncology	9 (50.0)
		All other GI Specializations	46 (47.9)
	Urban/rural location		
		Urban	132 (39.9)
		Rural	15 (25.0)
	Practice setting		
		Academic Center	49 (42.2)
		Non-academic Center	100 (36.2)
	Career stage		
		Fellows in training	21 (40.4)
		In practice 0–10 years	51 (38.6)
		In practice 11+ years	76 (36.7)
	Familiarity with the guideline		
		Familiar	67 (43.5)
		Unsure^a^	44 (37.0)
		Unfamiliar^b^	22 (26.8)

^a^Unsure means they are not clear on whether they are aware of the applicable guidelines or not

^b^Unfamiliar means providers are not well versed with current guidelines

### Impact of MSI/IHC results on timing of surgery and extent of resection

Physicians in academic centers were 2.41 times as likely as non-academic counterparts to agree abnormal testing results (MSI/IHC and/or germline) could impact decision processes regarding ECR (Table 5). Guideline familiarity was associated with the opinion surgery should not proceed until MSI/IHC results return as this could effect decisions regarding ECR. Only GI oncology sub-specialization and urban practice were associated with guideline familiarity (Supplementary table 1).

**Table 5:** Multivariable analysis of subgroup opinions relevant to the impact of MSI/IHC results on timing of surgery and extent of colonic resection

Statement for Evaluation	Demographic variables	Respondents answering “True” (Percentage)	Odds Ratio (95% confidence interval)
“Abnormal MSI/IHC/Germline testing results can affect extent of colonic resection.”	Gastroenterology (GI) subspecialty		
	General GI	175 (64.8%)	1.00
	GI Oncology Specializations	12 (80.0%)	1.04 (0.26, 4.25)
	All other GI Specializations	59 (71.1%)	0.98 (0.55, 1.74)
	Urban/rural location		
	Urban	210 (67.5%)	0.93 (0.50, 1.74)
	Rural	36 (63.2%)	1.00
	Practice setting		
	Academic Center	**80 (80.8%)**	**2.41 (1.25, 4.64)**
	Non-academic Center	168 (62.7%)	1.00
	Career Stage		
	Fellows in training	35 (76.1%)	1.33 (0.60, 2.97)
	In practice from 0 to 10 years	88 (72.7%)	1.57 (0.94, 2.61)
	In practice from 11+ years	122 (60.7%)	1.00
	Familiarity with the guideline		
	Familiar	115 (70.6%)	1.30 (0.73, 2.33)
	Unsure^a^	77 (62.6%)	0.91 (0.50, 1.66)
	Unfamiliar^b^	56 (65.9%)	1.00
“If MSI/IHC testing is ordered on CRC biopsy, surgery should wait to perform resection until after results have returned”	Gastroenterology (GI) subspecialty		
	General GI	80 (29.0%)	1.00
	GI Oncology Specializations	8 (53.3%)	1.54 (0.49, 4.89)
	All other GI Specializations	29 (31.9%)	1.04 (0.59, 1.83)
	Urban/rural location		
	Urban	107 (33.0%)	1.72 (0.84, 3.54)
	Rural	11 (18.6%)	1.00
	Practice setting		
	Academic Center	39 (36.8%)	1.39 (0.77, 2.51)
	Non-academic Center	79 (28.6%)	1.00
	Career stage		
	Fellows in training	13 (27.7%)	0.70 (0.31, 1.57)
	In practice from 0 to 10 years	44 (34.1%)	1.26 (0.76, 2.07)
	In practice from 11+ years	60 (29.0%)	1.00
	Familiarity with the guideline		
	Familiar	**65 (40.1%)**	**2.36 (1.25, 4.45)**
	Unsure^a^	35 (28.0%)	1.49 (0.76, 2.91)
	Unfamiliar^b^	17 (20.0%)	1.00

^a^Unsure means they are not clear on whether they are aware of the applicable guidelines or not

^b^Unfamiliar means providers are not well versed with current guidelines

### Physician opinion on ECR

Despite guidelines recommending ECR in LS, only 59.4% felt this was the preferred operation in patients < 50 (Table 6). 37.4% believed ECR was preferred in patients > 50. Academic practice settings were associated with belief ECR should be performed in young LS patients (74.8% opting for ECR).

**Table 6:** Multivariable analysis of factors associated with preference for performing total colectomy vs. segmental resection in Lynch Syndrome patients under age 50^a^

Variables	Number of respondents who prefer total colectomy with ileorectal anastomosis or proctocolectomy in the case of rectal cancer (percentage within each category)	Odds ratio (95% Confidence Interval)
Gastroenterology (GI) subspecialty		
General GI	153 (57.3)	1.00
GI Oncology Specializations	12 (80.0)	1.35 (0.33, 5.45)
All other GI Specializations	52 (61.9)	0.95 (0.55, 1.65)
Urban/rural location		
Urban	189 (61.2)	1.23 (0.68, 2.23)
Rural	28 (49.1)	1.00
Practice setting		
Academic Center	**74 (74.8)**	**2.20 (1.20, 4.03)**
Non-academic Center	144 (53.9)	1.00
Career stage		
Fellows in training	33 (71.7)	1.50 (0.70, 3.24)
In practice from 0 to 10 years	77 (63.6)	1.44 (0.89, 2.34)
In practice from 11+ years	108 (54.0)	1.00
Familiarity with the guideline		
Familiar	109 (67.3)	1.62 (0.92, 2.85)
Unsure^b^	64 (51.6)	0.90 (0.50, 1.61)
Unfamiliar^c^	46 (55.4)	1.00

^a^59.4% of total respondents surveyed prefer total colectomy or proctocolectomy for LS patients <50 years old, whereas 37.4% prefer the same operation in those age >50 years old (*p*<0.0001)

^b^Unsure means they are not clear on whether they are aware of the applicable guidelines or not

^c^Unfamiliar means providers are not well versed with current guidelines

## Discussion

We have uncovered barriers to LS screening which has provided insight into historically low MSI/IHC testing rates, particularly preoperatively. Understanding barriers is critical as failure of LS screening places both patients and family members at-risk. Study results reflect opinions of, and barriers experienced by, gastroenterologists. Gastroenterologists are important as they are frequently first to identify CRC during colonoscopy and can help facilitate LS identification, including preoperatively to assist surgical decision-making.

Importantly, there appears to be no consistent specialist who gastroenterologists feel is responsible for ordering MSI/IHC. Only 33.4% felt gastroenterologists should order MSI/IHC. Pathologists were believed most responsible followed by medical oncologists and surgeons. Such “diffusion of responsibility”, a sociopsychological phenomenon in which inaction may occur when multiple people are involved in decision-making, could potentially prevent testing, particularly preoperatively, and may help explain historically low testing rates^23^. Unless there are clear institutional protocols, with closed-loop communication between different specialists, respective physicians may incorrectly assume others are ordering testing. Previous research demonstrated that reflex (automatic) IHC and MSI testing institutional protocols are frequently not in place, particularly in community settings^24^. This could potentially allow diffusion of responsibility to negatively impact testing rates. Furthermore, a recent provider study, most of whom were non-gastroenterologists, revealed inconsistent LS identification and ad hoc testing practices with IHC ordered by a variety of providers^25^. Prior research demonstrated that even when institutional universal (all CRC cases) IHC testing was recommended, some might still not undergo testing^26^. Furthermore, if results were abnormal, genetic testing referral was inconsistent. This highlights assuring clear lines of provider responsibility for ordering, interpreting and acting on test results.

The most common testing barriers we uncovered were cost and lack of familiarity interpreting/applying MSI/IHC results. With regard to the later, pathology laboratories often provide interpretations of MSI/IHC results, which can help overcome this barrier. Nevertheless, lack of familiarity interpreting/applying results correlated with decreased guideline familiarity and highlights needs to enhance genomics education. Pertaining to cost, in many cases this may be only a perceived barrier as tumor testing is frequently, but not always (particularly in older patients), covered by insurance^27,28^. Furthermore, routine LS screening by MSI and IHC has been demonstrated as cost-effective with benefits for CRC patients and relatives^29^. Interestingly, a CRC patient survey revealed very positive attitudes toward LS tumor testing but a common barrier was cost concerns of additional testing and surveillance^30^. Clearly, testing coverage needs to be understood in each patient as out-of-pocket expense can prevent reliable testing. Insurance carriers not covering testing should be encouraged to do so given multiple benefits.

Lack of germline testing access and genetic counseling were also barriers. Non-academic and rural settings were associated with these barriers as well as with cost barriers. Suboptimal availability of genomic resources in these settings is a target for intervention. Decreased access to genetic specialists is a significant barrier to genetic testing and diagnosing LS^26^. Rural location was demonstrated as a barrier to genetics evaluation for hereditary breast cancer for multiple reasons including lack of awareness and distance of services^31^. Importantly, many genetic testing companies offer detailed interpretation of results and counseling services, which can help overcome these barriers. Furthermore, DNA sequencing cost has decreased in recent years^32^.

Timing of MSI/IHC result availability is important. Preoperative results effect decisions regarding germline testing and ECR. LS patients have increased risk of developing a 2nd CRC if colonic resection is limited. According to the U.S. Multi-Society Task-Force on Colorectal Cancer, to facilitate surgical planning, tumor testing should be performed on preoperative biopsy specimens, if possible^11^. According to American Society of Clinical Oncology, expediency in reporting biomarker results is important and need for evaluation is compounded by patient need to receive complete understanding of diagnosis and treatment plans going forward^33^. Additional benefit of preoperative testing is potential to perform prophylactic hysterectomy/bilateral salpingo-oophorectomy at time of CRC surgery in confirmed LS patients^34^.

Despite benefits of performing preoperative MSI/IHC, only 46.1% felt testing should be routinely conducted on colonoscopy specimens. If testing is performed only on surgical specimens, abnormal results return after the operation has been performed. In those non-routinely recommending preoperative testing, 43.7% also believed testing did not need to be routine on surgical resection specimens, which increases chances testing will never be performed. Many respondents, particularly routine colonoscopy specimen testers, also felt testing should be done concurrently on surgical specimens. Reasons for this are not entirely clear as this practice is not guideline supported. However, a potential explanation may be desire to assure concordance between colonoscopy and surgical resection specimens. It is important to address why providers might suggest MSI/IHC testing inconsistently (25–75% versus never or 100% of time). This may potentially reflect differences in patient-related factors (age, CRC family history, insurance coverage etc.) or practice settings (the same physician may work in multiple clinical settings with varying testing access).

Physicians in academic and urban settings were more likely to understand the importance of preoperative result availability. Guideline familiarity was associated with belief surgery should not proceed until MSI/IHC results return given potential affect on surgical decision-making. Guideline familiarity itself was associated with urban location and GI oncology sub-specialization. These findings likely reflect clustering of specialized services and potentially educational activities that may be available more frequently in academic and urban settings. Genomic educational programs in rural and non-academic settings can help overcome this issue.

GI fellows, those in early-career stages and those in non-academic settings felt waiting for MSI/IHC results would delay colon resection and therefore negatively impact outcome, thus preventing initial test ordering. Practitioners in non-academic settings believed waiting for germline testing results (if abnormal MSI/IHC) would delay resection and therefore negatively impact patient outcome. These findings potentially reflect a perceived or actual higher turnaround time for results in certain settings. This may also imply the complex multistep process (MSI/IHC followed by germline testing) can be a significant barrier.

Our survey also allowed assessment of opinions on colonic resection extent. Despite guidelines, only 59.4% felt in LS patients <50, ECR should be performed. Academic practice settings were positively associated with belief ECR should be undertaken. Assuring providers understand risks of synchronous and metachronous cancer is necessary. Notably, other factors may impact surgical resection decision-making including patient preference and potential effects on functional outcome^35^. However, a survey of colorectal surgeons revealed if pre-operative testing in early-onset CRC indicated LS, 84.9% would perform total colectomy^36^.

A potential study limitation is our survey response rate of 8.7%. Importantly, however, physician-based surveys may be less affected by non-response bias compared with other surveys due to higher homogeneity of knowledge, training, attitudes and behaviors than other groups^37^. Additionally, sensitive topics (in our survey, potentially adequacy of high-stakes genomics screening and assessing knowledge gaps) have been demonstrated to depress response rates, even in anonymous surveys^38^. Although we assessed which specialist gastroenterologists feel is responsible for ordering MSI/IHC, we do not have information on actual testing rates. Hence, another specialist may be ordering testing. However, given prior studies have shown low testing rates, this may not be occurring frequently. Finally, we do not have information on whether universal or reflex testing protocols are established at specific practices.

Study strengths include responses representing opinions from providers in multiple practice settings and career stages from across the U.S., which can increase generalizability compared to studies limited to specific settings. Furthermore, the distribution of academic versus non-academic settings was similar to distributions measured by the Association of American Medical Colleges, in which 19.5% and 12.3% reported current or prior academic faculty appointments respectively^39^. Additionally, the study focused on gastroenterologists who are important as they are often first to diagnosis CRC and can help identify LS preoperatively.

In conclusion, novel information has been obtained allowing better understanding of previously demonstrated low MSI/IHC testing rates and why results are infrequently available preoperatively. Testing cost, decreased availability of genomic services in rural and non-academic settings, guideline familiarity deficiencies and diffusion of responsibility regarding test ordering may be key barriers. Educating providers that testing costs have been decreasing, insurance frequently covers testing and that companies often provide result interpretation and counseling services can help circumvent many of these barriers. Importantly, a focus on underserved populations is required in which barriers, including financial/insurance coverage and access to specialized care, may be more pronounced.

Institutional Quality Assurance leadership should ensure systems-based universal testing protocols focusing on provider responsibility and timing of testing are implemented. Ultimately, the responsible specialist (pathologist, gastroenterologist, etc.) may depend on specific factors including expertise level interpreting results and the physician-patient relationship. However, roles should be clearly defined with closed-loop communication between different specialists to confirm testing is completed and results acted upon. Reflex (automatic) testing protocols, infrequently in place based on prior studies, can be beneficial, as this would prevent specific providers from being responsible for test ordering. However, the provider acting on results must be clearly designated particularly given that prior study demonstrated those with abnormal mismatch repair are inconsistently referred for germline testing. In any testing scenario, emphasis must be placed on institutional workflow protocols to assure both tumor analysis and germline testing are processed expeditiously and results available preoperatively to allow risk/benefit discussions on ECR. By removing ordering steps from the process, reflex testing may help streamline result availability. Similarly, requesting testing companies expedite germline analysis in the setting of a pending cancer operation can be beneficial.

Future studies are needed addressing testing/management practices in non-gastroenterologists, including surgeons, pathologists, oncologists, and geneticists. Furthermore, the divergent practice patterns we have demonstrated underscore needs to investigate other approaches to LS screening. New technologies including single up-front tumor next-generation sequencing are currently under investigation^40^. Another recent potential approach, which is discussed in the National Comprehensive Cancer Network guidelines specifically, includes performing multipanel (LS and other genes associated with hereditary CRC) germline testing in early-onset cases or those with a strong family history^41^. However, with adoption of any new technologies or approaches, well-defined protocols still need to be instituted assuring clear lines of responsibility regarding test ordering and interpretation, and efforts will be necessary to facilitate preoperative result availability.

## Study Highlights

### What is current knowledge

- Colorectal cancer tumor testing with microsatellite instability (MSI) and/or immunohistochemistry (IHC) to help identify Lynch syndrome (LS) is not performed routinely.
- Preoperative MSI/IHC testing on colonoscopy biopsy assists with germline genetic testing decisions and extent of colonic resection but is inconsistently conducted.
- Testing practices of gastroenterologists and barriers to testing, including preoperatively, are poorly understood.

### What is new here

- MSI/IHC testing barriers include cost, lack of familiarity interpreting results and genetic counseling access deficiencies.
- Testing barriers are clustered in rural and non-academic practice settings.
- No consensus exists among gastroenterologists regarding which specialist (gastroenterologist, pathologist etc.) should order MSI/IHC, which can prevent consistent testing.
- Testing on preoperative colonoscopy biopsy specimens is not routinely recommended which prevents availability of information to assist surgical decision-making.

## Electronic supplementary material

Supplemental Figure 1

Supplemental Table 1
