# Supplementary material for: Barriers to Lynch Syndrome Testing and Preoperative Result Availability in Early-onset Colorectal Cancer: A National Physician Survey Study
Source: Clin Transl Gastroenterol. 2018 Sep 20;9(9):185. doi: 10.1038/s41424-018-0047-y (PMC6148048; doi:10.1038/s41424-018-0047-y)
Supplement: Supplementary file 1 — Supplemental Figure 1 [file 41424_2018_47_MOESM1_ESM.docx]

Supplementary Figure 1. Survey Instrument

Early Onset Colon Cancer: Assessing Pitfalls in Lynch Syndrome Management

Start of Block: Consent Agreement

 1.| You are being invited to participate in an IRB approved study evaluating the management of early-onset colorectal cancer patients who may be at risk for Lynch syndrome, including physician practices regarding microsatellite instability testing (MSI).     If you agree to take part in this study, you will be asked to complete an online survey. We estimate that the survey should take less than 5 minutes to complete. You may not directly benefit from this research; however, we hope that your participation in the study will provide valuable patient care information that may help to change how early-onset colorectal cancer is managed.   We believe there are no known risks associated with this study; however, as with any online related activity the risk of a breach of confidentiality is possible, though remote.    The answers in this study will remain confidential. No identifiers (including but not limited to your name and place of work) will ever be linked to your survey submission, nor will this data ever be available to anyone other than the researchers directly involved in this study. Your participation in this study is completely voluntary and you can withdraw at any time.   **By selecting the statement below you are indicating that you are at least 18 years old, have read and understood this consent form and agree to participate in this study.  If desired, you can print a copy of this page for your records.**   **Participants will be eligible to enter a raffle for one of several $25 Amazon gift cards. Please see instructions at the end of the survey if you are interested in entry.**   *Please direct any questions to Dr. Jordan Karlitz, Tulane University School of Medicine - Department of Gastroenterology.*
 Email*: jkarlitz@tulane.edu*
 Phone*: (504) 988-5606*

- I have read the above statement and wish to begin this survey.

End of Block: Consent Agreement

Start of Block: Demographics

2.| Are you a current medical student, intern or resident?

- Yes
- No

3.| Which of the following best describes your medical specialty?

- Gastroenterology
- Colorectal Surgery
- General Surgery
- Surgical Oncology
- Pathology
- Other Medical Specialty (please specify) ________________________________________________

4.| Which of the following best describes the county where you primarily work? 

- Metro/Urban
- Non-Metro/Rural

If unsure, please click here (link to NCCN urban-rural continuum code). Please refer to 2013 and note that a county code of 1,2 or 3 is considered "Metro/Urban". All others should be considered "Non-metro/Rural".

5.| Which of the following best describes your practice setting?

- Multispecialty Private Practice
- Hospital employed physician
- Single specialty GI private practice
- University/Academic Center
- Veterans Affairs Facility

6.| Which of the following best describes your current career stage?

- Currently in fellowship
- 0-5 years post fellowship or terminal residency
- 6-10 years post fellowship or terminal residency
- 11-15 years post fellowship or terminal residency
- 16-20 years post fellowship or terminal residency
- I completed my fellowship or terminal residency more than 20 years ago

7.| What of the following best describes your GI subspecialty?

- GI Oncology
- Hepatology
- Inflammatory Bowel Disease
- Functional GI/Motility
- Advanced Endoscopy
- General Gastroenterology

End of Block: Demographics

Start of Block: Immunohistochemistry (IHC) & Microsatellite Instability (MSI) Testing Practices

8.| Given the following scenario:

 *A patient under 50 years old is diagnosed with Colorectal Cancer (CRC) on colonoscopy that will require treatment with colon resection.*
 
**In your clinical practice, approximately what percentage of the time do the following statements apply regarding testing for Hereditary Nonpolyposis Colorectal Cancer (HNPCC)/ Lynch Syndrome (LS)?**

|  | **0%** | **25%** | **50%** | **75%** | **100%** |
| --- | --- | --- | --- | --- | --- |
| Will plan to perform MSI and/or IHC testing for HNPCC/LS on tumor **biopsies taken during colonoscopy.** |  |  |  |  |  |
| Expect MSI and/or IHC testing for HNPCC/LS will be performed on **post-operative surgical resection specimen.** |  |  |  |  |  |

End of Block: Immunohistochemistry (IHC) & Microsatellite Instability (MSI) Testing Practices

Start of Block: Factors Affecting the Decision to Order Histologic and/or Genetic Testing

9.| Given the following scenario:

 *A patient under 50 years old is diagnosed with CRC on colonoscopy that will require treatment with colon resection.*
 
**For each of the following statements, please indicate whether each of the following prevents you or does not prevent you from ordering MSI and/or IHC testing.**

|  | **Prevents you from ordering MSI/IHC** | **Does not prevent you from ordering MSI/IHC** |
| --- | --- | --- |
| Lack of access to germline genetic testing if MSI/IHC abnormal. |  |  |
| Lack of access to genetic counseling at my facility. |  |  |
| Waiting for **MSI and/or IHC testing** results would delay colon resection and therefore negatively impact the patient's outcome. |  |  |
| Waiting for **germline testing** results (after initial MSI and/or IHC testing) would delay resection and therefore negatively impact the patient's outcome. |  |  |
| Lack of familiarity interpreting and applying the results from MSI and/or IHC testing. |  |  |
| Cost of MSI and/or IHC testing is prohibitive. |  |  |
| Ordering testing may adversely effect a patient's medical insurance status. |  |  |

10.| Who do you think is most responsible for ordering MSI and/or IHC testing?

- Gastroenterologist
- Surgeon (Colorectal Surgeon, General Surgeon, or Surgical Oncologist)
- Pathologist
- Medical Oncologist
- Medical Geneticist / Counselor

End of Block: Factors Affecting the Decision to Order Histologic and/or Genetic Testing

Start of Block: Timing of IHC and MSI Testing

11.| Given the following scenario:

 *A patient under 50 years old is diagnosed with CRC on colonoscopy that will require treatment with colon resection.*
 
**True or False: If MSI and/or IHC testing for HNPCC/LS is ordered on CRC biopsy, surgery should wait to perform resection until after results have returned.**

- True
- False
- Unsure

12.| Abnormal MSI and/or IHC or germline testing results suggesting HNPCC/LS can affect the decision making process regarding extent of colonic resection.

- True
- False

13.| In a patient diagnosed with HNPCC/LS in the following age groups, which of the following do you feel is the most appropriate surgical treatment?

|  | **Segmental Colon Resection** | **Total Colectomy with ileorectal anastomosis (or Proctocolectomy in the case of rectal cancer)** |
| --- | --- | --- |
| Less than 50 years old |  |  |
| 50 years or older |  |  |

14.| I am familiar with the guidelines (NCCN, ACG, etc.) regarding MSI and/or IHC testing for HNPCC/LS as applied to the above clinical scenario.

- Familiar
- Unfamiliar
- Unsure

End of Block: Timing of IHC and MSI Testing

Start of Block: Block 5

15.| Thank you for completing this survey! If you would like to be entered into a raffle for a $25 Amazon.com gift card. Please include your name and email address below. Please note, your answers will not be linked to your survey responses in any way.

- **Name** ________________________________________________
- **Email Address** ________________________________________________

End of Block: Block 5
