# Supplementary material for: Barriers to Lynch Syndrome Testing and Preoperative Result Availability in Early-onset Colorectal Cancer: A National Physician Survey Study
Source: Clin Transl Gastroenterol. 2018 Sep 20;9(9):185. doi: 10.1038/s41424-018-0047-y (PMC6148048; doi:10.1038/s41424-018-0047-y)
Supplement: Supplementary file 2 — Supplemental Table 1 [file 41424_2018_47_MOESM2_ESM.docx]

| **Supplementary Table 1.** Factors associated with self-reported familiarity with guidelines on tumor testing in CRC in patients under 50 years old, multivariable analysis^1^ | | | |
| --- | --- | --- | --- |
| Demographic Variables | | Number of the respondents reporting familiarity with guidelines (Percentage within category) | Odds Ratio  (95% Confidence Interval) |
| **Gastroenterology (GI) subspecialty** | |  |  |
|  | General GI | 114 (42.1%) | 1.00 |
|  | GI Oncology Specializations | **13 (86.75%)** | **8.20 (1.78, 37.82)** |
|  | All other GI Specializations | 35 (41.7%) | 0.96 (0.59, 1.56) |
| **Urban/rural location** | |  |  |
|  | Urban | **144 (46.2%)** | **1.84 (1.08, 3.14)** |
|  | Rural | 18 (31.0%) | 1.00 |
| **Practice Setting** | |  |  |
|  | Academic Center | 46 (46.0%) | 0.90 (0.54, 1.51) |
|  | Non-academic Center | 116 (43.1%) | 1.00 |
| **Career Stage** | |  |  |
|  | Fellows in training | 20 (43.5%) | 0.86 (0.45, 1.66) |
|  | In practice from 0-10 years | 51 (42.2%) | 0.91 (0.59 1.40) |
|  | In practice from 11+ years | 91 (44.8%) | 1.00 |

^1^The multivariable analysis controlled for the following variables: career stage, practice setting, urban/rural location, and GI subspecialty.
